# Obstetric referral processes and the role of inter-facility communication: the district-level experience in the Greater Accra region of Ghana

**DOI:** 10.4314/gmj.v56i3s.7

**Published:** 2022-09

**Authors:** Mary Amoakoh-Coleman, Kerstin Klipstein-Grobusch, Edem S Vidzro, Daniel K Arhinful, Evelyn K Ansah

**Affiliations:** 1 Department of Epidemiology, Noguchi Memorial Institute for Medical Research, University of Ghana, Legon, Ghana; 2 Julius Global Health, Julius Center for Health Sciences and Primary Care, University Medical Center Utrecht, Utrecht University, Utrecht, The Netherlands; 3 Department of Epidemiology and Biostatistics, School of Public Health, Faculty of Health Sciences, University of the Witwatersrand, Johannesburg, South Africa; 4 Center for Malaria Research, Institute of Health Research, University of Health & Allied Sciences, Ho, Ghana

**Keywords:** obstetric referrals, capacity, processes, outcomes, interfacility communication

## Abstract

**Objective:**

To describe the capacity of primary health care facilities to manage obstetric referrals, the reasons, and processes for managing obstetric referrals, and how an enhanced inter-facility communication system may have influenced these

**Design:**

Mixed methods comparing data before and during the intervention period.

**Setting:**

Three districts in the Greater Accra region, Ghana from May 2017 to February 2018

**Participants:**

Referred pregnant women and their relatives, health workers at referring and referral facilities, facility and district health managers.

**Intervention:**

An enhanced inter-facility communication system for obstetric referrals

**Results:**

Twenty-two facilities and 673 referrals were assessed over the period. The major reason for referrals was pregnancy complications (85.5%). Emergency obstetric medicines - oxytocin and magnesium sulfate (MgSO_4_) were available in 81.8% and 54.5% facilities, respectively, and a health worker accompanied 110(16.3%) women to the referral centre. Inter-facility communication about the referral occurred for 240 (35.7%) patients. During the intervention period, referrals joining queues at the referral facility decreased (7.8% to 0.0%; p=0.01), referrals coming in with referral notes improved (78.4% to 91.2%) and referrals with inter-facility communication improved (43.1% to 52.9%). Health workers and managers reported improvement in feedback to lower-level facilities and better filling of referral forms.

**Conclusion:**

Facilities had varying levels of availability of infrastructure, protocols, guidelines, services, equipment, and logistics for managing obstetric referrals. Enhanced inter-facility communication for obstetric referrals which engages health workers and provides requisite tools, can facilitate an efficient referral process for desired outcomes.

**Funding:**

This study was funded by the WHO/TDR Postdoctoral grant number B40347 to the NMIMR

## Introduction

Health systems may have different structures for handling referrals in general and obstetric referrals in particular. Medical and non-medical reasons for referrals may vary from pregnancy complications and health system factors to even personal ones.^1^–^3^ Obstetric complications are often unpredictable. They can occur at any time during the pregnancy with bad outcomes without necessary interventions.^1^ Referral to higher levels of care with personnel, infrastructure and logistics to manage the complication may become necessary.

Obstetric referrals, whether self-referrals or institutional, often occur during delivery and may often be emergency rather than elective.^3^–^5^ The sole aim of an efficient (obstetric) referral system is to assure good outcomes for both mother and newborn, but many factors such as patient factors and the structure of the referral system.^6^–^8^ influence the realisation of this goal, An efficient referral system amongst other things prevents delays.^7^ and so requires that the decision to refer is appropriate, patients accept the decision, transportation to referral facility occurs promptly, especially during emergencies and there is adequate communication between the referring and referral facilities.^6^,^7^ These efforts should be augmented by efficiently mobilising key resources for emergency obstetric and neonatal care (EmONC) services.

In Ghana, a referral policy and guidelines document developed by the Ministry of Health (MOH) in 2012 stipuates the standard processes for general referral 9, while the national Safe Motherhood Protocol (SMP) describes the levels of care for pregnant women and processes for obstetric referrals. The policy identifies inefficiencies in the referral system, which include patients bypassing the first level of care, lack of standard procedures, delays in referral and non-use of referral forms. It prescribes the referral process to include completion of the referral form and immediate onset of communication about the referral between the two facilities. The policy is unrestrictive and adaptable by agencies under the MOH. The Ghana Health Service (GHS) practices a referral system that starts from the community-based health planning and ser-vices (CHPS) to the sub-district level, where there are community clinics and health centres, eventually ending up at the district hospital. Most institutional obstetric referrals to the district hospital are expected to follow this bottom-up approach, although, as noted in the referral policy, some bypass this channel, endangering the gate-keeper mechanism.^9^

We aimed to describe the capacity of primary health care facilities to manage obstetric referrals, the reasons and processes for managing obstetric referrals, and how an enhanced inter-facility communication system may have influenced these.

## Methods

### Study design

We conducted a formative evaluation using a mix of a cross-sectional study, pre and post-intervention analysis, and qualitative study from May 2017 to February 2018. This study is nested within a bigger study that looked at the role of an enhanced inter-facility communication system in the processes and outcomes of obstetric referrals. The details of the methodology for the bigger study have been described in another paper. 10

### Study setting

The Greater Accra region has 20 administrative metropolises, municipalities, and four rural districts. The study occurred in Ga West, semi-urban, rural Ada East and Ningo-Prampram. Ga West and Ada east have district hospitals. Ningo-Prampram district has a polyclinic as the highest-level public facility; thus, referrals are sent to a private hospital, which is included in this study, though most referrals go outside the district.

***Participants***: There were three groups of participants in this study:

1. Pregnant women referred from lower levels of care (health centre, polyclinic, community clinic and CHPS compound), within the district to the district hospital (referral may or may not end up in delivery but not self-referrals).
2. Health workers and managers at all levels of care within the district directorate.
3. Pregnant women attending antenatal clinics in the district and their family members.

***Description of intervention*:** An intervention package of an enhanced inter-facility communication system was implemented for 4 months after 5-month baseline data collection. The intervention package consisted of:

1. Training health workers on interfacility referral communication, including sharing patient information and accurate documentation using referral notes.
2. Provision of communication tools (working phones and call credits for health workers to facilitate calls and text messaging).
3. Designating someone or a team responsible for interfacility communication in referral facilities (includes the specialist in the referral facility) and linking all such agents or teams to all the facilities within a district. These teams had monthly meetings to review obstetric referrals.
4. Strengthening and enforcement of feedback mechanisms between referring and referral facilities.

### Data collection and variables

*Quantitative*: We conducted a facility audit to assess the capacity of the facilities to handle obstetric referrals at baseline. We also collected data on monthly referral statistics from the referring and referral facilities in the districts throughout the study period. Finally, we collected data on some process indicators for each obstetric referral through records review using a questionnaire throughout the study period.

***Qualitative*:** FGDs and IDIs were conducted for data on the processes of maternal referrals. There were six focus group discussions at baseline, two sets in each district, one for health workers and another for pregnant women and their spouses/ partners/ caregivers. There were three sets after the intervention period, one for each district with health workers since they directly benefited from the intervention. District, facility and obstetric/maternity unit heads were interviewed at baseline and after the intervention. Discussions and interviews were conducted in English, Ga and Twi and tape-recorded. Table 1 shows the description of the variables explored. A facility audit was done at the beginning of the study. Facility statistics records review and weekly observation data were collected during the baseline (here refers to the period before intervention was rolled out) and during the intervention period. FGDs and IDIs were done at the beginning of the baseline and end of the intervention period.

**Table 1:** Description of variables for the study

Quantitative: Facility audit, Participant records review & statistics	Qualitative: FGDs/ IDIs & Observations
**Human resource** Doctors Nurses & Midwives Pharmacists Laboratory staff	FGDs (Women) Reasons for referral Expectations from HW during referral Perception about referrals Acceptance of referral Challenges & Recommendations
**Logistics, Protocols & registers** Phones National referral policy & guidelines and other protocols Referral note booklets Referral registers Obstetric medication stocks	FGDs (Health workers) Reason for referral Involvement of patients in referral Pre-referral management Tools for inter-facility communication Use of Referral notes Influence of intervention package Challenges & recommendations
**Services** Vaginal delivery Caesarean section Manual removal of placenta PMTCT of HIV/ AIDS Resuscitation of newborn Repair of vaginal tears Blood transfusion Laboratory services	IDIs Reasons for referral Factors influencing referral Involvement of patients in referral Transportation Communication Referral protocols and guidelines Referral notes Influence of intervention package Challenges and recommendations
**Statistics (monthly per facility)** Total antenatal clinic (ANC) attendance Total deliveries Total Antepartum hemorrhage cases Total postpartum hemorrhage cases Total pre-eclampsia/ eclampsia cases Total referrals in Total referrals out Total maternal mortality	Observations (weekly per facility) Timing of referrals *(morning, afternoon, night)* Timing of referral *(antenatal, delivery, postnatal)* Urgency of referrals HW accompanying emergencies Transportation of referrals Inter-facility Communication Availability of doctor during referral
**Participant record review** Reason for referral Emergency or not Referral note provided Need for admission at referral center Mode of transport Interfacility communication Patient goes directly to referral center Time to reach referral center	

### Data analysis

Descriptive analysis was done using frequencies and proportions for all variables for quantitative data. Bi-variate analysis using the chi-squared (χ2) test was used to investigate the influence of the intervention, before and during the intervention, with the detection of significance set at a two-sided p<0.10 due to the relatively short period of implementation of the intervention package.

Data analysis was done using IBM SPSS Statistics for Windows, Version 20.0. Armonk, NY: IBM Corp. Qualitative data were audio/ hand recorded, transcribed verbatim, and all Twi and Ga responses were translated into English. Content analysis was carried out for patterns and emerging themes related to the study objectives.

### Ethical approval

The study was approved by the Noguchi Memorial Institute for Medical Research (NMIMR) Scientific and Technical Committee (STC) and the Institutional Review Board (NMIMR-IRB CPN 072/16-17) as well as the Ghana Health Service (GHS) Ethical Review Committee (GHS-ERC:11/01/2017). Permission was obtained from the Greater Accra Regional Health Directorate, the participating district health directorates, and the heads of the selected facilities. Written informed consent was obtained from all participating women.

## Results

### Primary health care facility capacity to manage obstetric referrals and statistics

A total of twenty-two facilities were assessed: three hospitals, one polyclinic, eight health centres, two community clinics and eight CHPS compounds. Apart from one hospital and one privately owned clinic, the other health facilities were all government-owned. Ten (45.5%) facilities received referrals from lower-level facilities. From the facility audit at baseline, most facilities (86.4%) work 24 hours per day, while 13 (59.1%) have a functional facility phone. Only 45.5% of these phone numbers were available and known to other facilities. One hospital had another phone in the maternity unit. A functional ambulance was present in 3 (13.6%) facilities, which referred patients must pay (often upfront) to use. Most facilities (86.4%) had paper-based medical records with only one facility insisting on referral notes from referred patients before registering them.

Regarding documents, all the facilities had the Standard Treatment Guidelines, (11)50.0% had the national referral policy and guidelines, 18(81.8%) had the safe motherhood protocol and 19 (86.4%) each had the referral note booklet and referral registers. Generally, the bigger facilities had more staff, with doctors concentrated mainly in the hospitals. Emergency obstetric medicines such as oxytocin and magnesium sulfate (MgSO_4_) were respectively available in 18(81.8%) and 12(54.5%) facilities, with stock outs experienced within six months prior to the study. Nine out of the ten referral facilities conducted haematological and malaria tests. Seven facilities provided ultrasonographic services, while only one facility (hospital) could carry out clotting tests. All three hospitals provided caesarean section services, but only two provided blood transfusion services.

There were 60,082 antenatal clinic visits and 10,399 deliveries. The total referrals-out of facilities were 1,393, with the majority (81.9%) made to the hospitals. Total cases of antepartum haemorrhage, postpartum haemorrhage and gestational hypertension and its complications were 110, 106 and 369 respectively. Maternal mortality over the study period was nine.

### Reasons and Processes for Obstetric referrals

For the nine-month study period, a total of 1,386 obstetric referrals were attended to in the three district hospitals. A total of 673 obstetric referrals from nine-teen (19) lower-level facilities met our inclusion criteria and were analyzed for referral processes.

From the records review of referred women, most referrals to the district hospital were made by midwives (94.5%), and 51.9% were emergency referrals. Major reason for referrals was pregnancy complications (85.5%). Table 2 shows how different measures of the referral process performed at baseline and during the intervention period. Following the intervention, significant improvement occurred in client acceptance of referrals, referrals with referral notes, those receiving requisite first aid and going straight to the referral center.

**Table 2:** A description of quantifiable processes of obstetric referrals before and during the intervention package

Variable	Category	Frequency (%)/ Mean (SD) N=673	Baseline N (%)	Intervention N (%)	p-value
**Covered by Health Insurance**	Yes	662 (98.4)	252 (38.1)	410 (61.9)	0.62
**Age (Mean/ SD)**		28.1 (6.3)	28.3 (6.5)	28.0 (6.1)	0.43
**Referring health worker**	Doctor	8 (1.2)	0 (0.0)	8 (100.0)	0.07
	Midwife	636 (94.5)	246 (38.7)	390 (61.3)	
	Nurse	13 (1.9)	3 (23.1)	10 (76/9)	
	Other	16 (3.6)	8 (50.0)	8 (50.0)	
**Is referral emergency**	Yes	349 (51.9)	140 (40.1)	209 (59.9)	0.30
**Reason for referral**	Complication	576 (85.5)	207 (35.9)	369 (64.1)	<0.01
**Need for referral explained to client**	Yes	570 (84.7)	226 (39.6)	344 (60.4)	0.07
**Client agrees with referral**	Yes	603 (89.6)	253 (42.0)	350 (58.0)	<0.01
**Patient requires first aid**	Yes	337 (50.1)	155 (46.0)	182 (54.0)	<0.01
**Patient receives first aid**	Yes	364 (54.1)	155 (42.6)	209 (57.4)	0.01
**Patient accompanied by health** **worker**	Yes	110 (16.3)	40 (36.4)	70 (63.6)	0.67
**Patient arrives with a referral note**	Yes	469 (69.7)	195 (41.6)	274 (58.4)	0.01
**Patient requires admission at referral** **facility**	Yes	549 (81.6)	224 (40.8)	325 (59.2)	<0.01
**Patient admitted**	Yes	539 (79.5)	221 (26.6)	314 (58.7)	<0.01
**Inter-facility communication**	Yes	240 (35.7)	63 (26.3)	177 (73.7)	<0.01
**Patient comes straight to referral** **facility**	Yes No	460 (68.4)	139 (30.2)	321 (69.8)	<0.01

### Characteristics of the majority of weekly referrals to the three hospitals

**Figure 1:**
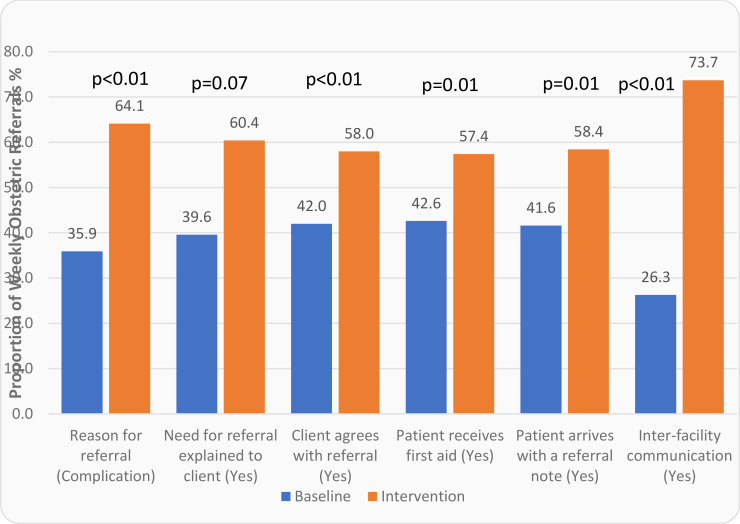
Comparison of obstetric referral process variables for baseline and intervention periods in the three districts

Non-participant observation of 85 weekly-referral patterns in the three hospitals showed a significant positive relationship between emergency referrals and whether a caesarean section was required and a negative association between emergency referrals and whether a health worker accompanied the referrals (p<0.01). Figure 1 shows how some process variables changed during the intervention period, and all changes were significant.

1. The reasons and processes for obstetric referrals emerged mainly from the qualitative data. Our findings can be presented under three themes:
  - Reasons for referrals and factors considered (including decision-making and patient involvement)
  - Process of effecting the referral (including meeting guidelines requirement, transportation, and inter-facility communication)
  - Challenges and recommendations to improve referrals

### Reasons for referrals and factors considered

Major reason for referrals to higher levels of care mentioned by participants during the IDIs and FGDs, was severity of pregnancy complications (mainly high blood pressure, pre-eclampsia and eclampsia, abnormal presentation, placenta previa, sickle-cell patient, preterm labor, anemia, etc.).

“*We refer cases that are beyond us like B/P 140/90, pre-eclampsia, placenta previa, post-date pregnancies, preterm deliveries” (FGD, health worker, District 3, baseline)*

Other reasons were the absence of infrastructure and/or equipment for services such as caesarean sections, delivery, blood transfusion and ultrasound scan services.

Almost all participants in the women/family FGDs reported experience of referrals during pregnancies and that most referrals are during delivery and tend to be emergency. The choice of referral facility is often based on the availability of a specialist and the facility's capacity to manage the condition, an example being NICU services required for preterm deliveries. Other factors considered are patient preference, cost of care and distance to the facility.

*“We refer because the hospital is the highest facility that can manage such cases. We ask the patient's preference... and we also take into consideration, the distance and cost”. (FGD, health worker, District 3, baseline)* In emergency situations, decision is made by the health worker in the best interest of the patient.

### Process of effecting the referral

*Acceptance/Refusal of Referral*

Health workers reported patients' refusal of referrals both at baseline and during the intervention. Reasons for refusal included fear and apprehension, financial considerations, unknown environment, unfriendly health workers at the referral center, amongst others

*“Sometimes they don't want to go at all because they are used to receiving treatments here”. “Sometimes they would agree to go but after leaving the facility they divert to somewhere else. Some also go to prayer camps”. (FGD, health worker, District 2, baseline)*

Some refuse referrals mainly due to fear of severe condition and dire consequences.

*“They sometimes refuse referral because they are afraid. ..... They even tell you a relative died when referred so they will not go. Some of them panic with increase in their BP (blood pressure)”. (FGD, health worker, District 3, baseline)*

*“I have seen two patients referred and they died so if I am referred, that rings in my mind and I get worried” (FGD, woman, District 3, baseline)*

Sometimes, they refuse because they believe the primary level facility can take care of their condition.

*“I was referred because they said I cannot deliver by myself, so I need CS. However, when I got to the hospital, I delivered by myself without CS”. (FGD women District 3, baseline)*

Financial considerations include the cost involved in the transfer, transportation, and cost of care at the higher level of care. Credit facilities available to them at the referring facility where they are known may not be available at the referral centre.

*First aid before referral*

Health workers reported that often first aid is not required, especially in non-emergency situations, but pain relief or IV fluids, where necessary, should be provided. In emergencies, some first aid is provided to stabilize patients. This includes resuscitation using IV fluids in the case of bleeding, giving the loading dose of MgSO_4_ for pre-eclampsia, anticonvulsant for eclampsia and administration of Hydralazine, Nifedipine or other antihypertensive drugs for hypertension.

*Use of Referral Forms/Notes*

The national and facility protocols are the only guidelines used when referring patients. Health workers know that the national referral guidelines require referral notes for referrals to facilitate work at the receiving facility.

During baseline and intervention periods, referral notes were not consistently provided to patients in non-emergency and antenatal referrals. Most health workers indicated that they always give referral notes before referring patients. However, during the FGDs for women at baseline, they reported they are often not given any referral notes. Heads of facilities and health workers of some referral facilities also corroborated the point that often, referred patients come in without referral notes. When probed further, some health workers said that it is mainly due to the unavailability of referral forms and self-referrals when they do not give referral notes. Health workers preferred to put referral information in the ANC booklets. They also complained that were provided; the referral forms were not adequately filled with the requisite information. However, during the intervention period, the provision of better-filled referral notes by health workers improved.

*“They did not give me any referral note to send to Prampram, they just told me that from today onwards go to Prampram. When you get there (the referral center), they ask you a lot of questions, so if they give us a note that will be helpful. (FGD, women, District 2, baseline)*

*“It (the intervention) has improved our work because formerly we had gaps in our referral forms but now, we fill it appropriately because of the training given to us” (FGD, health worker, District 1, intervention)*.

Some health workers said their facilities run photocopies of the referral forms before they run out, and others who did not have stocks designed their own forms like the original GHS referral forms to use.

Health workers and managers, however, mentioned that in some cases patients do not show up with referral notes because they hide them when they get to the referral facility, both during the baseline and intervention periods.

*“Yes, we give them, but some of them take it and put them in their bags”. (FGD, health worker, District 2, baseline)*

*“Yes, we always do (i.e., give them referral notes), but sometimes the patients hide them and say they have not been given any. They refuse to show it because they want a different opinion at the referral facility”. (FGD, health worker, District 3, intervention)*

### Role of the enhanced Inter-facility Communication on the processes and outcomes of referral

1. At baseline, health workers expressed concern about poor inter-facility communication. Receiving facilities complained that referring facilities do not call them before patients show up, which can be particularly challenging. The referring facilities stated that they make calls to receiving facilities, but their calls go unanswered. Poor communication network and unavailability of facility phones or phone credit were mentioned as reasons for poor inter-facility communication. Health workers reported they sometimes call other facilities or personal phones of staff in other facilities using their personal phones. Some districts have WhatsApp platforms where they communicate with each other.
2. Information communicated to the referral facilities includes the referral indication and management given in some cases. After the intervention, the health workers said they were happy about the improvements. In FGD with the health workers, some said: *“With the help of the referral team meetings, we identified a private facility that was sending very bad cases of referrals, so we followed up and realized that they were operating with untrained health professionals”. (FGD, health worker, District 3, intervention)*

Others mentioned that the intervention helped them give feedback to lower-level facilities like the health centres on referrals, especially with the need to avoid referral delays. Facility managers, heads of maternity units and district health managers appreciated the impact of the intervention on the referral processes. They stated that they had reduced referrals of conditions they could handle but previously would have referred due to the training received. They can also better fill out the referral forms and provide better referral feedback. The provision of phones to facilities has particularly improved inter-facility communication, although sometimes the facilities do not pick up their calls. Most facilities now call before referrals are sent over, which has been extremely helpful, allowing for rapid response to the cases on arrival at the referral centre. They also identified challenges with obstetric referrals and made recommendations to address them within the facilities. Facility heads and health workers are now updated on referrals and plan to continue having review meetings even after the study.

*“The intervention has had a great impact on our work, and we urge management to continue providing the phone credits for us to be able to communicate easily with other facilities”. (FGD, health worker, District 1, intervention)*

*Transportation for referrals*

Participants reported unreliable transport systems in their facilities. Many facilities did not have functional ambulances. There is usually only one available in the district, and patients have to pay for fuel before utilization. Most patients cannot afford the high cost of using the ambulance, with some resorting to using taxis. In some situations, the ambulance charge is almost ten (10) times the taxi fares.

*“In our facility, we ask the relative to arrange for transport themselves. The ambulance would take as much as 200 cedis but the taxi can take 20 cedis for a trip to Korle-bu”. ((FGD, health worker, District 2, intervention)*

During emergencies, health workers sometimes arrange for ambulances or taxis to take patients to the referral facilities. Sometimes, relatives arrange for the transportation themselves. Occasionally, patients in labour are transported on motorbikes.

In non-emergency cases, patients arrange their own means of transport, usually on commercial vehicles.

Health workers sometimes accompany the patient to the referral facility for emergency cases. However, since most facilities have limited staff, they cannot accompany referred patients. Almost all the health workers confirmed that they follow up on referred pregnant women through visits or calls.

Challenges and recommendations to improve referrals

1. Health workers and managers listed some challenges they face with obstetric referrals and proffered some recommendations
2. to deal with them. These are summarized in Table 3.

**Table 3:** Challenges with obstetric referrals and recommendations (from facility audit and IDIs)

Challenges with referrals	Recommendations to improve referrals
**Transportation: no ambulances, so use of taxis, motorbikes, trotro, etc.).** **High cost**	Improve transport: functional ambulances for facilities/districts -with free services especially in emergency
**Client refuses referrals: they go elsewhere and return later with complications.** **Fear that referral means bad consequences.**	Educate clients: need for referrals and consequences of not accepting referral.
**Financial challenges: cost of transport, staying in a far-off place and of** **management of patients**	Allocate funds in districts to support referrals. NHIS to work effectively at all facilities Pregnant women prepare financially for unforeseen emergencies.
**Poor inter-facility communication about referrals: no calls, or staff are** **reluctant to pick up calls.** **Non-functional facility phones; health workers use their personal** **phones without reimbursement; poor network**	**Continuous education of staff to call referral centers for emergency referrals.** **Provide tools for communication (phones, credits) for health workers.** **Improve network service**
**Inadequate staffs: increase work load and inability to accompany emergency** **referrals.**	Improve staffing at all levels.
**Delayed referral decisions and late arrivals, with more complications.** **Appropriate first aid not administered**	Early referral decisions. Educate clients. Provide first aid to stabilize patients.
**No referral notes**	Provide referral notes for all, especially emergencies. Adequate and consistent provision of Referral booklets.
**Referral centers have NO BEDS, and refuse referrals**	Check the appropriateness of referral facility before referral, including the availability of bed for patients if required.
**Lack of logistics (drugs and tools) for service**	Provide logistics for all facilities
**Poor road networks to access facilities**	Improve road networks especially outside the urban areas.

## Discussion

### Main results

Facilities had varying levels of availability of infrastructure, protocols, guidelines, services, equipment and logistics for managing obstetric referrals. Emergency obstetric medicines such as oxytocin and magnesium sulfate (MgSO_4_) were not present in all facilities, while blood transfusion service was provided in two of the three hospitals. About half of obstetric referrals were emergency referrals, with the majority referred by midwives and related to delivery. Complications of pregnancy were the main reasons for referral.

Just as in other studies, most referrals were transported using private commercial transportation and most emergency referrals were not accompanied by a health worker to the referral center.^1^,^11^

During the intervention period, there was an improvement in referrals with inter-facility communication. Patient Concerns about referrals related to fear of the outcome of care and financial implications, amongst others. Health workers' concerns about referrals related to the refusal of referrals and poor inter-facility communication.

### Implications for service delivery

With only one referral hospital per district, there is a heavy workload on these hospitals, potentially leading to long waiting times at the referral center.^12^, ^13^ District hospitals manage about two-thirds of all obstetric complications in Ghana.^1^ In our study, they delivered 64.9% of births in the three districts, which is higher than the 52% they were found to handle nationwide in one study.^1^ The capacity of these hospitals to manage pregnancy complications, however, appears to be limited, since the referrals out of the hospitals were 18.9%, far above what was found for district hospitals (9%) and tertiary and regional hospitals (under 1%) in a national referral assessment.^1^ The same study documents that staff at this level of care worked to the best of their ability under sub-optimal conditions. Many studies have documented pregnancy complications as major reasons for obstetric referrals 12, 14 and reflect the limited capacity of healthcare providers at the lower levels and the need for improved referral systems. Improving the capacities and skills of frontline providers will reduce the number of referrals due to complications as alluded to by providers. Non-medical reasons such as lack of staff with requisite training and lack of infrastructure and logistics also accounted for some referrals, and this has also been documented in other studies.^14^, ^15^ Continuous training of staff and provision of infrastructure for basic emergency obstetric care (EmOC), will enable providers at lower levels of care better manage some of the complications, so that referrals occur only when necessary. Unfortunately, varying levels of readiness to provide EmOC at lower levels of care in Ghana have been demonstrated 1, and gaps need to be practically addressed sustainably.

Midwives oversee obstetric care in lower-level facilities and are trained to identify and manage risk and refer when appropriate. To reduce pregnancy complications, midwives in Ghana receive regular training to improve their competencies and skills. However, these skills or competencies cannot yield desired outcomes if midwives are not provided with the infrastructure, equipment, and logistics to deliver the needed service. In the absence of an enabling environment, midwives will focus on managing low-risk pregnancies and refer high-risk ones.^1^,^16^,^17^

Many clients refuse referrals for various reasons, including fear of worse outcomes, financial constraints and unfriendly workers at the referral centre.^1^ In a study that examined non-completion of referrals back to outpatient specialty clinics, some reasons for refusal of the referrals included patient choice, physical or social barriers and communication failure.^18^ Patients have challenges, and the need for referral must be communicated to facilitate their acceptance.^1^ The connection midwives establish with women at the community level and also through the antenatal care process potentially facilitates the communication of risk to women and subsequent uptake of referrals.^17^,^19^ Being made a part of the decision-making process is desirable and helps patients understand that the referral is in their best interest for the best outcomes.^20^–^22^

Inter-facility communication is a very important part of the referral process and a key tool for monitoring the effectiveness of obstetric referrals, as it ensures appropriate and timely care for the client. 6 Lack of feedback to the referring facilities is a barrier to the functionality of referral systems.^19^ The requirement of the national referral protocol is not always adhered to from our findings, with logistical challenges cited as main reason. Our intervention which was tailored to meet the needs of health workers based on baseline FGDs and IDIs, was well received and made some significant improvement on some of the processes of referral. Provision of logistics (such as functional phones with call credits) and routine review of referrals should be prioritized if obstetric referrals are to achieve their intended results.

*Limitations and strengths*

The use of mixed methods in studying this topic is a strength of this paper as it allowed us to interrogate the nuances of the functionality of obstetric referrals at the district level from key stakeholders' perspectives. Our inability to work in a purely urban setting where complex dynamics of referrals between public and private facilities exist means our results may not truly reflect obstetric referral processes in such settings.

## Conclusion

Enhanced inter-facility communication for obstetric referrals, which engages health workers and provides requisite tools, can facilitate an efficient referral process for desired outcomes.
